# Arthropod and oligochaete assemblages from grasslands of the southern Kenai Peninsula, Alaska

**DOI:** 10.3897/BDJ.5.e10792

**Published:** 2017-01-12

**Authors:** Matthew L. Bowser, John M. Morton, John Delton Hanson, Dawn R. Magness, Mallory Okuly

**Affiliations:** 1 U.S. Fish & Wildlife Service, Kenai National Wildlife Refuge, Soldotna, Alaska, United States of America; 2 Research and Testing Laboratory, Lubbock, Texas, United States of America

**Keywords:** metagenomics, Arthropoda, exotic earthworms, Illumina MiSeq

## Abstract

**Background:**

By the end of this century, the potential climate-biome of the southern Kenai Peninsula is forecasted to change from transitional boreal forest to prairie and grasslands, a scenario that may already be playing out in the Caribou Hills region. Here, spruce (Picea × lutzii Little [*glauca* × *sitchensis*]) forests were heavily thinned by an outbreak of the spruce bark beetle (*Dendroctonus rufipennis* (Kirby, 1837)) and replaced by the native but invasive grass species, *Calamagrostis canadensis* (Michx.) P. Beauv. As part of a project designed to delimit and characterize potentially expanding grasslands in this region, we sought to characterize the arthropod and earthworm communities of these grasslands.

We also used this sampling effort as a trial of applying high-throughput sequencing metabarcoding methods to a real-world inventory of terrestrial arthropods.

**New information:**

We documented 131 occurrences of 67 native arthropod species at ten sites, characterizing the arthropod fauna of these grasslands as being dominated by Hemiptera (60% of total reads) and Diptera (38% of total reads). We found a single exotic earthworm species, *Dendrobaena octaedra* (Savigny, 1826), at 30% of sites and one unidentified enchytraeid at a single site. The utility of high-throughput sequencing metabarcoding as a tool for bioassessment of terrestrial arthropod assemblages was confirmed.

## Introduction

### Background

By the end of this century, the potential climate-biome of the southern Kenai Peninsula is forecasted to change from transitional boreal forest to prairie and grasslands (Scenarios Network for Arctic Planning and EWHALE lab 2012). This may be happening presently in the Caribous Hills region on the southern Kenai Peninsula between Tustumena Lake and Kachemak Bay, where Lutz spruce (Picea × lutzii Little [*glauca* × *sitchensis*]) forests were heavily thinned by a massive outbreak of the spruce bark beetle (*Dendroctonus rufipennis* (Kirby, 1837)) in the 1990s (Berg et al. 2006, Boucher and Mead 2006, Sherriff et al. 2011). Between 1987 and 2000, basal area of Lutz and white spruce >12.7 cm diameter-at-breast height decreased by 87% in this region (Boucher and Mead 2006).

Following this outbreak, the native but invasive herbaceous species *Calamagrostis canadensis* (Michx.) P. Beauv. and *Chamerion angustifolium* (L.) Holub increased in abundance (Boucher and Mead 2006). Although initial recruitment of spruce seedlings following this outbreak was sufficient to restock these forests (Boggs et al. 2008), large areas were subsequently burned in the 1990s and 2000s, potentially killing tree seedlings and further contributing to a transition from spruce forest to grassland.

Of the fauna of northern grasslands, arthropods are among the most abundant, diverse, and ecologically important (see Shorthouse and Larson 2010). Though Steppe bison (*Bison priscus* Bojanus, 1827) and mammoths (*Mammuthus primigenius* (Blumenbach, 1799)) existed on the Kenai Peninsula in the Pleistocene (Klein and Reger 2015) and Dall sheep (*Ovis dalli* Nelson, 1884) inhabit the Kenai Mountains, no large, mammalian grass-grazing herbivores remain in the Caribou Hills, leaving arthropods as the most ecologically important herbivores in grass- and forb-dominated habitats.

With the exception of Teraguchi et al. (1981), who sampled terrestrial arthropods from a *C. canadensis*-dominated grassland in Interior Alaska but did not obtain species identifications, we were able to find scant data on the arthropod communities of this habitat type in Alaska. We sought to characterize the arthropod assemblages of this potentially expanding grassland community on the Kenai Peninsula.

Lumbricid earthworms are relatively recent arrivals to Alaska translocated from the Palearctic by human activities (Hendrix and Bohlen 2002). They are at present more common near roads than in more remote areas on the Kenai Peninsula (Saltmarsh et al. 2016). As potential agents of change that can alter soil properties when introduced into new areas (Hale et al. 2005, Frelich et al. 2006, Hale et al. 2006, Holdsworth et al. 2007), we were interested in documenting the current distribution of earthworms in Kenai grasslands. We also wanted to determine the identities of the worms because the effects of earthworm invasions are dependent on the species composition of earthworm assemblages (Hale et al. 2005, Frelich et al. 2006).

### Metabarcoding as a tool for assessing terrestrial arthropod assemblages

The investigator seeking to characterize assemblages of arthropods or of other diverse groups is currently presented with a wide and growing range of options for obtaining species identifications including traditional, specimen-based, morphological identifications; Sanger sequencing of individual specimens using DNA barcodes (Hebert et al. 2003) or similar short marker sequences; High-throughput sequencing (HTS) of individual specimens targeting short marker sequences (Shokralla et al. 2014, Meier et al. 2015); PCR-based HTS of mixed environmental samples from homogenized specimens (Hajibabaei et al. 2011) or preservative fluid (Hajibabaei et al. 2012); and PCR-free HTS (Zhou et al. 2013, Shokralla et al. 2016).

High-throughput sequencing metabarcoding methods have been advocated for biomonitoring of arthropod communities because they have the potential to be quick and comparatively inexpensive (Hajibabaei et al. 2011, Baird and Hajibabaei 2012). Several recent studies (Hajibabaei et al. 2012, Carew et al. 2013, Elbrecht and Leese 2015, Gibson et al. 2015, Aylagas et al. 2016) have demonstrated the utility of metagenomic HTS for characterizing mixed samples of invertebrates.

Obtaining correct species identifications from HTS methods requires a well-curated library of sequences from identified specimens (Hajibabaei et al. 2011, Dowle et al. 2015). Toward this end the first and second authors have been contributing arthropod sequences from specimens in the entomology collection of the Kenai National Wildlife Refuge to the BOLD database (Ratnasingham and Hebert 2007) beginning in 2007. Sikes et al. (in press) greatly expanded this work, sequencing specimens from the University of Alaska Museum's entomology collection, contributing to an Alaska DNA barcode library with the explicit purpose of enabling identification of Alaskan terrestrial arthropods by DNA barcoding.

In this small project we applied HTS metabarcoding methods to a real-world inventory with a vision of applying similar methods to future biomonitoring efforts.

## Materials and Methods

### Study area and study design

Our study area was a 37,790 ha union of major fire polygons south of Tustumena Lake on the southern Kenai Peninsula. This included the 1994 Windy Point Fire, 1996 Crooked Creek Fire, 2005 Fox Creek Fire, and 2007 Caribou Hills Fire.

**Figure 1.**
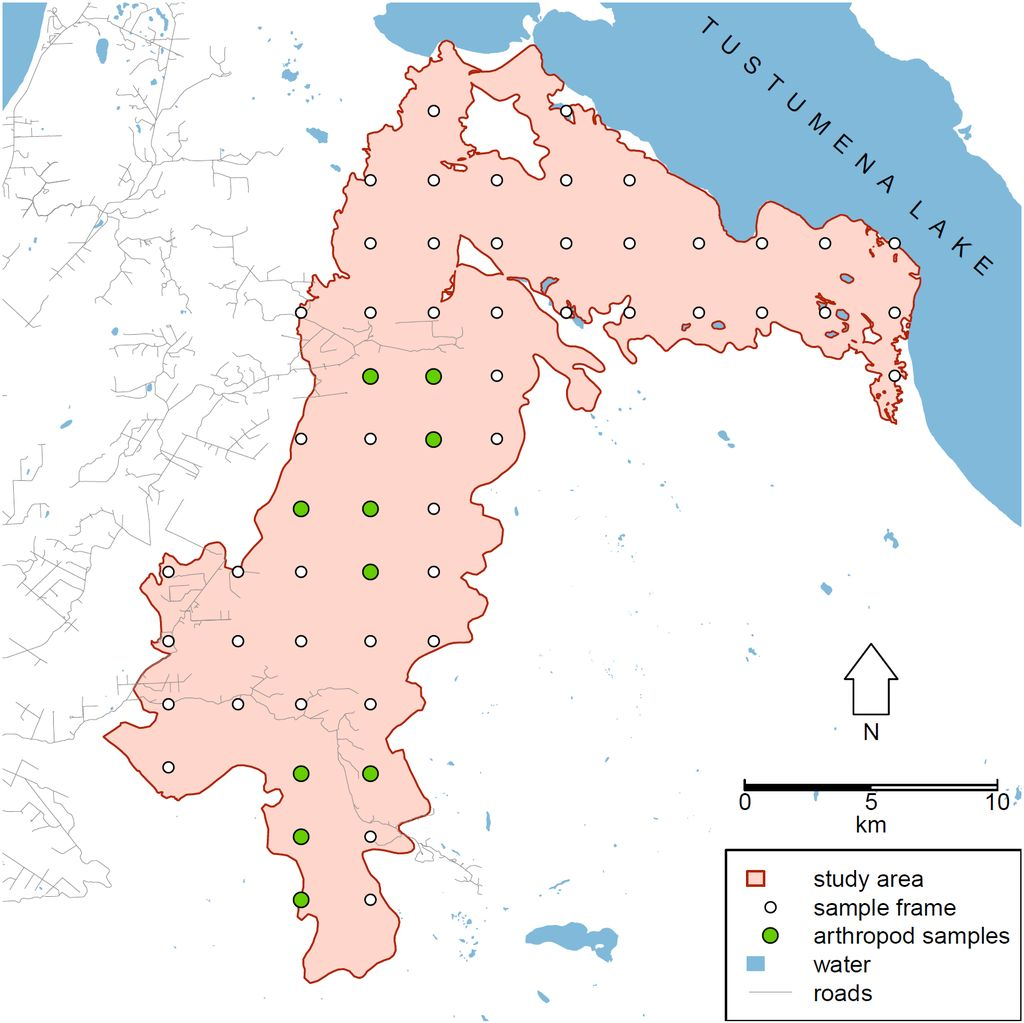
Map of the study area, southern Kenai Peninsula, Alaska.

Within this area, we chose as a sample frame to use centroids of the 250 m pixels from the Alaska eMODIS product (Jenkerson et al. 2010​), selecting every 12^th^ pixel in both north-to-south and east-to-west axes, making a grid of 58 points spaced at 3 km intervals (Fig. 1).

### Field methods

Sampling sites were accessed using a Bell 206B Jetranger on July 18-19, 2015. Only when a site was determined from the air to be a non-wetland grassland as defined by Viereck et al. (1992) did we land.

All plant species within a 5.64 m radius, 100 m^2^ circular plot centered on the plot coordinates were recorded. Plants that could not be identified in the field were collected.

Earthworms were collected at each plot using methods similar to those of Saltmarsh et al. (2016). First, vegetation was removed from a small area within the plot using clippers, then a 50 cm × 50 cm aluminum quadrat frame was set on the ground. We searched through surface litter and organic material for earthworms by hand, then we extracted additional earthworms with a liquid mustard solution of 40 g yellow mustard seed powder (Monterey Bay Spice Company, Watsonville, California, http://www.herbco.com) in 3.8 L water (Lawrence and Bowers 2002). Earthworm specimens were collected into Uni-Gard -100 propylene glycol antifreeze.

At ten sites we collected a single sample of arthropods by sweeping the same 5.64 m radius plot in under five minutes using a BioQuip™ model 7112CP net with 30.5 cm diameter, approximately 24 × 20 per inch mesh BioQuip™ model 7112CPA net bag and a BioQuip™ model 7312AA 30.5 cm extension handle. Sweep net samples were placed in 250 ml Nalgene^®^ vials filled with Uni-Gard -100 propylene glycol antifreeze, then stored in a -23°C freezer.

### Laboratory methods

Plant specimens were identified in the laboratory using the keys of Hultén (1968), Welsh (1974), Tande and Lipkin (2003), and Skinner et al. (2012).

We identified earthworm specimens visually using the key of Reynolds (1977). Worm specimens were deposited in the entomology collection of the Kenai National Wildlife Refuge (coden: KNWR) and specimen data were made available via Arctos (http://arctos.database.museum/). One small worm that we could not identify morphologically we submitted for DNA barcoding via a LifeScanner kit (http://lifescanner.net/).

Arthropods were separated from vegetation and debris by hand under a dissecting microscope. At the same time, all athropods were tallied and coarsely identified, generally to orders but sometimes to families, genera, and species that could be quickly identified by sight. We made no attempt to account for the varying sizes of different arthropods.

Specimen data (Table 1) were entered into Arctos, where all data including site photographs from this project are available via an Arctos project entitled "Southern Kenai Peninsula grassland study" (http://arctos.database.museum/ProjectDetail.cfm?project_id=10002178). Corresponding records were entered into GenBank as BioSamples (BioProject PRJNA321553, https://www.ncbi.nlm.nih.gov/bioproject?term=PRJNA321553).

Samples were shipped in propylene glycol to RTL Genomics in Lubbock, Texas for sequencing. Upon arrival, samples were removed from propylene glycol and rinsed with 100% ethanol. Ethanol rinse was decanted and enough 100% ethanol was added to the container to cover the arthropods. Samples were stored in Ethanol for 21 days. Samples were then rinsed in PBS, then 400 μl of PBS was added to the sample and the sample was ground using an Omni Tissue Homogenizer. Extraction was performed using MoBioPower soil extraction kit with an overnight incubation at 37°C. To elute the sample 50 μl of prewarmed elution buffer was added to the column membrane and incubated at room temperature for 2 min, then spun down. The elutate was place back on the column and incubated another 2 min, then spun down.

We used the forward primer *mlCOIintF* (GGWACWGGWTGAACWGTWTAYCCYCC) and reverse primer *HCO2198* (TAAACTTCAGGGTGACCAAAAAATCA). These primers used previously by Leray et al. (2013) and Brandon-Mong et al. (2015), yielding a 313 bp fragment from the Cytochrome oxidase I DNA barcoding region. Primers were ordered with a 5' extension following the Illumina 2-step amplicon protocol. Samples were amplified in 25 μl reactions with Qiagen HotStar Taq master mix (Qiagen Inc, Valencia, California), 1 μl of each 5 μM primer, and 1 μl of template. Reactions were performed on ABI Veriti thermocyclers (Applied Biosytems, Carlsbad, California) under the following thermal profile: 95°C for 5 min, then 25 cycles of 94°C for 30 sec, 54°C for 40 sec, 72°C for 1 min, followed by one cycle of 72°C for 10 min and 4°C hold. Following amplification, reactions were separated on 2% agarose gels (Egels; Invitrogen, Carlsbad, California) and added to the next reaction based on band strength. A second amplification was performed using primers based on the Illumina Nextera PCR primers as follows: Forward - AATGATACGGCGACCACCGAGATCTACAC-[i5index]-TCGTCGGCAGCGTC and Reverse - CAAGCAGAAGACGGCATACGAGT-[i7index] GTCTCGTGGGCTCGG. Following amplification reactions were separated on 2% agarose gels (Egels; Invitrogen, Carlsbad California) and pooled equimolar based on band strength. Pools were run through a Qiagen Qiaquick gel column (Qiagen Inc, Valencia, California) and eluted in 50 μl, followed by small fragment removal using Agencourt AMPure XP beads at 75% (BeckmanCoulter, Indianapolis, Indiana). The pool was run on a Fragment Analyzer (Advanced Analytical, Ankeny, Iowa) and quantified using Qubit (Invitrogen, Carlsbad, California). The pool was prepared for sequencing using Illumina MiSeq V3 chemistry following manufacturer instructions, sequenced for 500 flows (2x250) and demultiplexed by on board software.

Sequence data were submitted to GenBank's Sequence Read Archive (BioProject: PRJNA321553).

Total molecular lab processing cost was $1,115 ($111.50 per sample) and sequencing results were delivered 68 days after samples had been received by RTL Genomics.

### Library construction and metagenomic analysis

For the present study, we constructed an Alaska vicinity reference library by downloading publicly available COI data from BOLD on January 20-21, 2016, entering the search terms "Arthropoda[tax] Alaska[geo]" and similarly structured searches for arthropod sequences from the Yukon Territory, British Columbia, Chukot Autonomous Okrug, and Kamchatka Krai, yielding an initial library of 236,830 records including 6,677 unique species name strings.

A metagenomic analysis was performed using the cloud-based Galaxy platform (Giardine 2005, Blankenberg et al. 2010, Goecks, J. et al. 2010), generally following the simple metagenomics pipeline of Brandon-Mong et al. (2015) as an example.

Where one of a pair of reads had a read length less than 250 bp, these were filtered out using R version 3.2.2 (R Core Team 2015) and the ShortRead package (Morgan et al. 2009), then the resulting FASTQ files were uploaded to Galaxy. FASTQ files were merged using PEAR version 0.9.6.0 (Zhang et al. 2013), accepting default settings. Merged sequences were converted to sanger format using FASTQ Groomer version 1.0.4 (Blankenberg, Daniel et al. 2010). We used Filter by quality version 1.0.0 from the FASTX-toolkit (Gordon 2010) to filter reads by quality using default settings (cut-off=20, percent=90). Filtered reads were converted to FASTA file format using Galaxy's FASTQ to FASTA converter version 1.0.0 (Blankenberg et al. 2010). Chimeric sequences were removed using VSearch chimera detection version 1.9.7.0 (Rognes et al. 2016), accepting default settings. Sequences were then dereplicated using VSearch dereplication version 1.9.7.0 (Rognes et al. 2016), accepting default settings except that cluster abundances were written to the output files. Clustering was performed using VSearch clustering version 1.9.7.0 (Rognes et al. 2016), CD-HIT method with minimum identity set at 0.90.

Identifications were improved iteratively. First, initial identifications were obtained by querying the cluster centroids against our reference library using VSearch search version 1.9.7.0 (Rognes et al. 2016), accepting default parameters except that minimum similarity was set at 0.90. This yielded identifications at varying levels of taxonomic resolution because many identifications in our library were coarse identifications at the resolutions of genera, families, and orders. We chose to retain all library records, even those missing species names because we wanted to represent the assemblages as well as possible, including taxa for which we could not obtain Linnaean names with currently available information.

For all library records that were matched by our queries and that lacked species names we added identifications by submitting them to BOLD's Identification Request service and updating our library records with any identification improvements. In cases where no species names were available, we constructed provisional names incorporating BOLD BIN URIs (Ratnasingham and Hebert 2013), for example "Anthomyiidae sp. BOLD:AAG2469" corresponding to BIN BOLD:AAG2469. In cases where our library sequences closely matched multiple Linnaean species names on BOLD, the corresponding BIN generally including multiple Linnaean species, we again reverted to BIN resolution identifications or appropriate Linnaean names where these were available, e.g. "*Simulium venustum* complex" corresponding to BIN BOLD:AAA4264.

For cluster centroids that were not matched by our library, we queried these against the BOLD database using the bold() function from the bold package for R, version 0.3.5 (Chamberlain 2016). Where we found problematic records, especially those tagged as contaminated, we removed these from our library. The resulting library included 236,837 records (Table 2). Where matches were found among publicly available BOLD records, we downloaded these sequences and added them to our library, resulting in inclusion of a small number of sequences from Alberta, Manitoba, Northwest Territories, Ontario, Prince Edward Island, and Quebec.

The VSearch step was repeated using the improved library and the resulting occurrence data were submitted to Arctos as observation records (GUIDs: UAMObs:Ento:235609–UAMObs:Ento:235739).

We repeated the VSearch search identification step against our improved library using the same parameters. For the purpose of reporting species occurrence we exlcuded all clusters where read counts were four or less and all clusters where the VSearch search similarity values were less than 0.91. Clusters matching human COI were dropped.

## Results

### Vegetation

**Figure 2.**
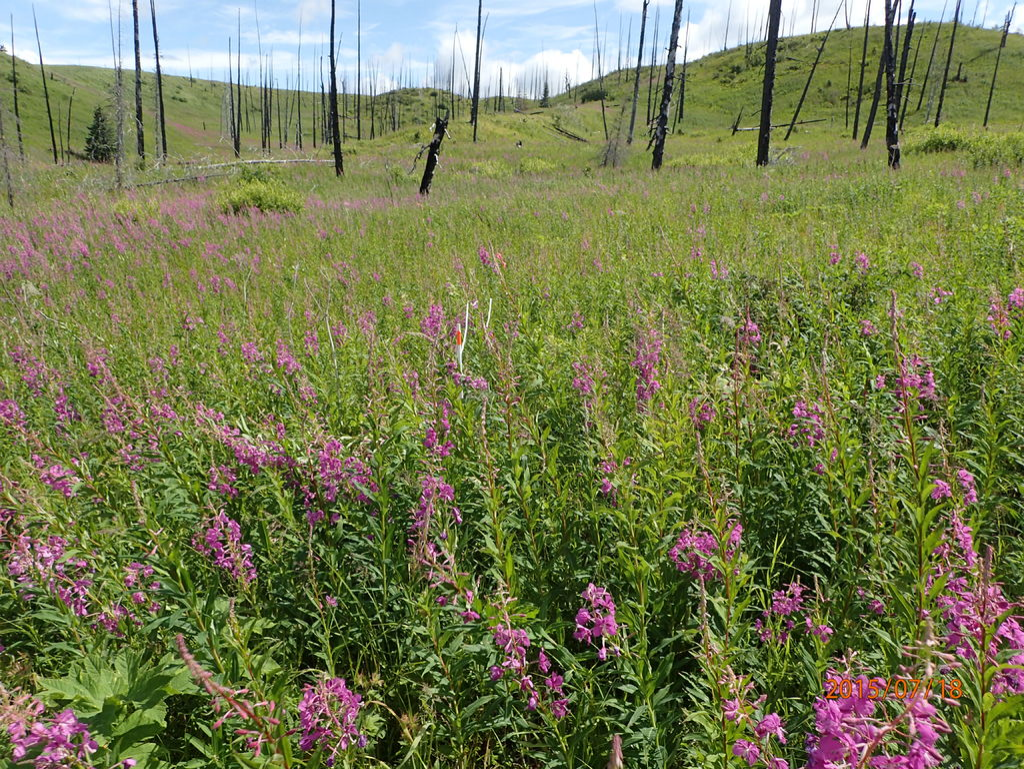
Study site 73F (60.1055°N, 151.1806°W), a site characteristic of our study area dominated by *Calamagrostis canadensis* and *Chamerion angustifolium*, photographed on July 18, 2015. Note the fire-scarred remains of a Lutz spruce forest that was culled by an extensive spruce bark beetle outbreak in the 1990s and subsequently burned in 2007.

The ten plots were dominated by herbaceous plants, characterized by *Calamagrostis canadensis* (Michx.) P.Beauv. and *Chamerion angustifolium* (L.) J. Holub, species present at all sites (Fig. 2). *Streptopus amplexifolius* (L.) DC., *Sanguisorba canadensis* L., *Veratrum viride* Aiton, *Dryopteris expansa* (C. Presl) Fraser-Jenk. & Jermy, *Geranium erianthum* DC., and *Lupinus nootkatensis* Sims were found at six or more of the ten sites (Table 5, Suppl. material 1).

### Oligochaetes

At three sites (30% of sites) we detected a single earthworm species, *Dendrobaena octaedra* (Savigny, 1826). From another site a single specimen (Arctos GUID: KNWR:Ento:10822) was identified as an enchytraeid based on its COI sequence (BOLD Process ID: MOBIL1272-16). This sequence differed from all other sequences on BOLD, founding a new BIN (BOLD:ADC0663) with a nearest neighbor identified as *Mesenchytraeus orcae* Eisen, 1904 (pairwise-distance: 3.51%). Collection data for oligochaetes are provided in Suppl. material 2.

### Arthropod morphological identifications

**Figure 3.**
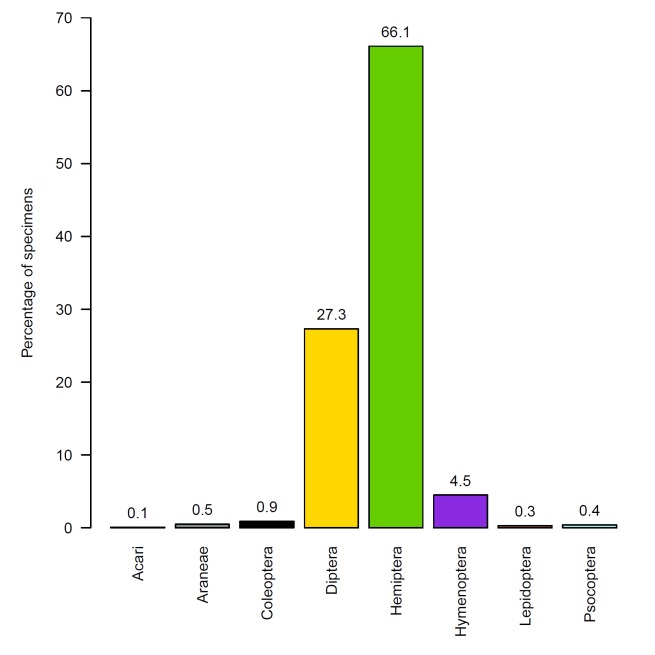
Percentages of total specimens collected by orders identified by sight.

Based on tallies of the sample contents by sight identifications, the sweep net samples contained 22–325 (mean=103, SE=26) individuals per sample, a total of 1,029 specimens (Table 3). Identifications were made at varying taxonomic resolutions: 416 specimens only to orders, 580 specimens only to families, 18 only to genera, and 15 to species. Eight orders (Fig. 3), 27 families, six genera, and two species were represented. Complete occurrence data based on sight identifications are included as supplementary material (Suppl. material 3).

The samples were dominated by Hemiptera (66% of total specimens), especially the family Cicadellidae (25% of total specimens), and by Diptera (27% of total specimens). Hymenoptera represented only 4.5% of the specimens while Acari, Araneae, Coleoptera, Lepidoptera, and Psocoptera each represented less than 1% of specimens.

### Arthropod metagenomic identifications

**Figure 4.**
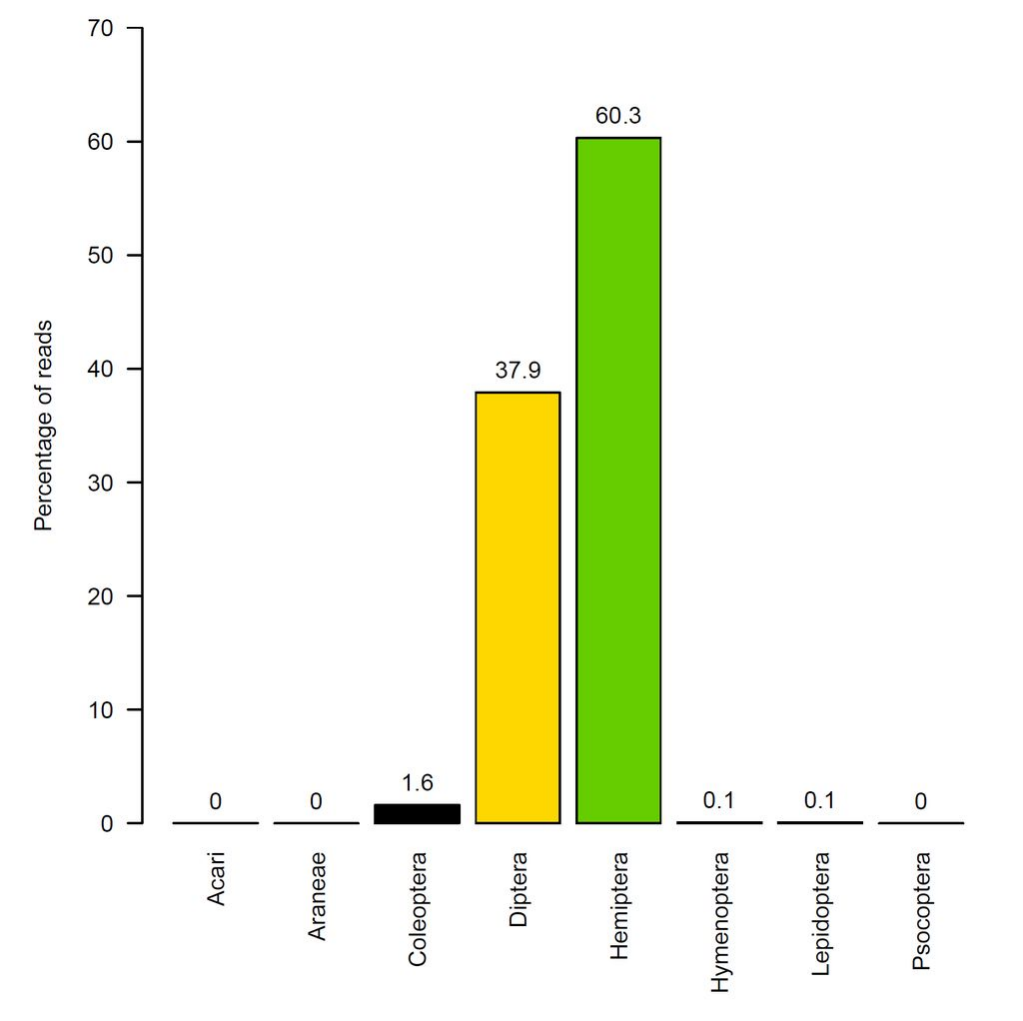
Percentages of the total numbers of reads by orders.

Sequencing yielded 30,672–54,228 reads per sample (mean=45,194, SD=7,601), a total of 451,941 reads. At the end of analysis and filtering steps, 391,316 identified reads were included in the occurrence data, 26,066–47,402 reads per sample (mean=39,132, SD=7,064) representing seven orders (Fig. 4). Data for all identified clusters are included as supplementary material (Suppl. material 4).

Of the 391,316 reads included in the occurrence data, these were dominated by Hemiptera (60%) and Diptera (38%). Coleoptera made up 1.6% of the reads while Araneae, Hymenoptera, Lepidoptera, and Psocoptera each included less than 1% of reads. No reads of Acari were identified.

Including provisional names, the metagenomic analysis yielded 67 unique taxon names (Table 4), 5–19 names per sample (mean=13.2, SD=4.7, see Suppl. material 5. The identifications represented 63 unique BINs. Four of the matched taxa lacked corresponding BINs.

Of the two species identifications we were able to make by sight, both were detected and identified by the metagenomic analysis. *Misumena vatia* (Clerck, 1757) was detected in the same sample in both the sight identifications and the metegomic data. *Lauxania shewelli* Perusse & Wheeler, 2000 was recorded at six sites in the sight identifications and detected at five of these same six sites in the metagenomic analysis.

Scrutiny of the remaining sequences that did not match anything in our reference database revealed a total of ten reads of human sequences from three sites.

## Discussion

### General characterization

Within the *Calamagrostis*-dominated grasslands of the Caribou Hills region we documented an entomofauna dominated by Hemiptera and Diptera, comparable to the general composition of sweep net samples collected in a Montana grassland by Spafford and Lortie (2013). Teraguchi et al. (1981) collected arthropods from a recently burned, *Calamagrostis*-dominated grassland similar to the sites we sampled in the Caribou Hills, but meaningful comparisons between our datasets are problematic due to the lack of details provided by Teraguchi et al. (1981).

We collected a similar number of specimens in ten 100 m^2^ sweep net samples over two days as Teraguchi et al. (1981), who collected 1,112 arthropod specimens in 18 0.43 m^2^ samples over three months from a recently burned *Calamagrostis canadensis* grassland in the vicinity of Fairbanks, Alaska. Their field collecting method of sampling arthropods from vegetation using a D-Vac vacuum insect collector would have been expected to obtain results generally comparable to our sweep net sampling method (Schotzko and O'Keeffe 1989) although vacuum collectors do tend to collect a greater numbers of individuals (Schotzko and O'Keeffe 1989, Buffington and Redak 1998, Doxon et al. 2011), lower biomass (Doxon et al. 2011), smaller size classes (Doxon et al. 2011), and similar (Doxon et al. 2011) to higher (Buffington and Redak 1998) species diversity compared to sweep net sampling per unit effort. The overall composition of the communities collected by Teraguchi et al. (1981) cannot be directly compared to ours because they did not provide the numbers of individuals collected for each order.

Teraguchi et al. (1981) recognized “about 265” morphospecies, four times more than the 67 taxon names yielded by our metagenomic analysis. This difference may have been at least partially due the much longer temporal sampling window of June through August used by Teraguchi et al. (1981), where they would have been able to collect arthropods species having varying seasonal phenologies. Some of the difference is attributable to the ability of the D-Vac vacuum to collect a greater diversity of arthropods than sweep net sampling, but most of the difference is likely due to the identification methods used. Few species, even rare species, would have been missed by morphological identifications; our metagenomic methods likely failed to detect rare species as was the case for Hajibabaei et al. (2012). With the exception of the Coleoptera, of which they collected none, Teraguchi et al. (1981) found a greater diversity of species within all orders of arthropods compared to our data. Particularly notable was the Hymenoptera, of which Teraguchi et al. (1981) recognized over 140 morphospecies; we found five. However, Teraguchi et al. (1981) obtained no species identifications using recogized scientific names, greatly limiting the usefulness of their results. In contrast, our methods yielded identifications that can be related to described species or at least recognizable molecular operational taxonomic units (MOTUs) (Blaxter et al. 2005).

Although our arthropod sampling methods captured only a portion of the total arthropod fauna that would have been present, our results portrayed a reasonable snapshot of at least the fauna present on vegetation. All arthropods we documented are believed to be native to Alaska.

### Comments on selected taxa

The single exotic earthworm species we collected, *Dendrobaena octaedra*, present at 30% of sites in our study area, was already known to be widespread on the Kenai Peninsula. This species was found at 70% of sites sampled on the Kenai National Wildlife Refuge, adjacent to our present study area, by Saltmarsh et al. 2016. A parthenogenetic, epigeic species, *D. octaedra* is believed to be spread easily by vehicle tires (Cameron et al. 2007), but it causes little change in soil properties as compared with earthworm assemblages that include anecic and endogeic earthrworms (Hale et al. 2005, Frelich et al. 2006). Based on our finding of only a single exotic earthworm species, a species known to have little effect on soils, exotic earthworms are likely to contribute relatively little to changes in grasslands of the southern Kenai Peninsula in the near future unless anecic or endogeic earthworms become established.

We assume that the single enchytraeid we collected was native because enchytraeids are widespread and diverse in southern Alaska (see Timm 1999).

The chrysomelid beetle *Altica tombacina* was documented at two sites in the metagenomic analysis. Review of the notes associated with the specimen records on Arctos showed that these had been larvae when collected and so would have been unlikely to be identifiable based on morphology. *Altica tombacina* is to be expected in the study area, having been described from the Russian River vicinity (Mannerheim 1853) about 70 km to the northwest.

The two staphylinid beetles seen in our samles were missed by our metagenomic methods likely due to their generally small size, primer bias, or a combination of these two reasons.

One of the more frequently detected species was *Coenosia impunctata* Malloch, 1920 (Diptera: Muscidae), found at seven sites. This species, described from Mount Katmai, Alaska (Malloch 1920), is distribubted from the Aleutian Islands to British Columbia based on data in BOLD.

Pipunculidae, specialist parasitoids on Cicadellidae and Delphacidae that are easly recognized at the family level, were seen in only two of the samples, but reads were detected in six samples in the metagenomic analysis, representing three species. At least some of these reads almost certainly came from pipunculid larvae within their cicadellid hosts.

Cicadellidae were well represented in our metagenomic data both in terms of read abundance and diversity, consistent with the high abundance and diversity of cicadellids documented from Canadian grasslands (Hamilton and Whitcomb 2010).

Of the Cicadellidae, the most common was *Sonronius dahlbomi* (Zetterstedt, 1840), detected at eight out of ten sites. According to Beirne (1956), this is a locally common species ranging from Alaska to Newfoundland and Labrador.

An entity bearing the provisional name of "*Euscelis monodens* sp. nov" (BIN BOLD:ACG7815) was the next most common cicadellid, detected at five sites. This provisional species is currently represented on BOLD by 15 specimens from British Columbia and the Yukon.

Delphacidae, herbivores of graminoids previously found by Bowser (2009) in 18% of sweep net samples from all habitat types on the Kenai National Wildlife Refuge, adjacent to our study area, were conspicuously absent from these *Calamagrostis*-dominated post-fire grassland samples.

It was noteworthy that *Nabis* (Nabidae) specimens were seen in the samples at four sites, but these were not detected by the metagenomic analysis despite these being some of the largest specimens in the samples, representing a significant portion of the material by body mass.

*Irbisia sericans* (Stål, 1858) (Hemiptera: Miridae), which we detected at one site, had previously been documented from *Calamagrostis*-dominiated grassland on the southern Kenai Peninsula where they had caused chlorosis of *Calamagrostis* leaves and stunting of the plants (McKendrick and Bleicher 1980).

Human COI sequences in our data may have been due to contamination in our processing steps, but these may alternatively have come from human blood within biting flies collected in our samples. Biting flies (*Simulium* or *Symphoromyia*) were detected in all three samples where human sequences were detected (see Suppl. material 4).

### Metabarcoding as an identification method

The overall metagenomic results were consistent with our accounting of the specimens by eye, consistently portraying a community dominated by Hemiptera and Diptera. Our metagenomic methods under-represented the Araneae, Hemiptera, Hymenoptera, Lepidoptera, and Psocoptera while over-representing Coleoptera and Diptera relative to the proportions of specimens, likely due to primer bias during the PCR step. This is consistent with the experience of Brandon-Mong et al. (2015) and Aylagas et al. (2016), who documented some PCR bias using the same *mlCOIintF*/*HCO2198* 313 bp region but found that it generally performed well over a broad range of invertebrate taxa compared to other regions that they tested.

To date, the purpose of most studies of involving HTS metabarcoding of arthropods has generally been to test and refine these methods (see Hajibabaei et al. 2011, Hajibabaei et al. 2012, Carew et al. 2013, Brandon-Mong et al. 2015, Elbrecht and Leese 2015, Aylagas et al. 2016). Ours is among the first studies to apply these methods to a real-world inventory effort (but see Gibson et al. 2015).

Our metabarcoding methods yielded timely (about 80 days including lab processing, shipping time, and analysis steps) and relatively inexpensive identifications ($US 1,115 for 131 sample × taxon identifications, $US 8.51 per identification). This is considerably more expensive than the < $US 0.40 chemical cost per identification of Meier et al. (2015) and the < $US 1 cost per morphological identification cost of Meierotto and Sikes (2015), but in both of these cases there would have been additional time and expense required for curating and archiving individual arthropod specimens. In contrast, our methods required only that vegetation and debris be separated from arthropods prior to forwarding samples to the metagenomics lab, a step that took < 1 hr. per sample.

There is an obvious trade-off between curating individual specimens for long-term deposition in an institutional repository and homogenizing specimens for HTS. Archiving individual specimens would have the potential to yield the most information as the specimens can be photographed, identified, and sequenced individually, and the specimens remain available for use in subsequent work. Rare and small species, easily missed by our HTS metagenomic methods, would be more likely to be detected using specimen-based, morphological methods.

However, processing and identification of thousands of specimens is time-consuming (Marshall et al. 1994). In addition, many specimens may remain unidentified if they are immature, damaged, or members of groups for which taxonomic expertise is unavailable. Metabarcoding can be more taxonomically comprehensive than morphological methods (Ji et al. 2013), providing identifications over a broad range of taxa.

A non-destructive metabarcoding method (Hajibabaei et al. 2012) would appear to be ideal for rapid bioassessments, providing rapid identifications while leaving specimens intact, but most arthropod metabarcoding studies to date have relied on extraction of DNA from homogenized tissue. We chose this method simply because it was already available as a service from a metagenomics lab.

## Conclusions

We documented a native grassland arthropod fauna dominated by Hemiptera and Diptera. We found a single, epigeic, exotic earthworm species, but earthworms are unlikely to significantly alter these grassland communities unless additional exotic earthworms become established. We also demonstrated the usefulness of high-throughput sequencing metabarcoding as a tool for bioassessment of terrestrial arthropod assemblages.

## Supplementary Material

Supplementary material 1 Vegetation data Data type: occurrences
Brief description: Observation-based occurrences of vascular plant species. Dates are given in ISO 8601 format.
File: oo_106140.xlsx Matthew L. Bowser, John M. Morton

Supplementary material 2 Earthworm specimen data Data type: occurrences
Brief description: Earthworm specimen data. Dates are given in ISO 8601 format.
File: oo_106202.xlsx Matthew L. Bowser, John M. Morton

Supplementary material 3 Arthropod sight identification occurrences Data type: Occurrence
Brief description: Arthropod specimen counts by sight identification and sample. Columns labeled KNWR:Ento:10838–KNWR:Ento:10847 are GUIDs of corresponding records on Arctos.
File: oo_90113.xlsx Matthew L. Bowser

Supplementary material 4 Cluster identification data from metagenomic analysis Data type: Occurrences
Brief description: GUID: Arctos globally unique identifiers for the arthropod samples. Cluster label: illumina labels of centroid sequence clusters. Read count: cluster read counts. Process id: BOLD process IDs for matched database sequences. Similarity: similarity value from VSearch search. BIN: BOLD Barcode Index Numbers. Nucleotides: cluster centroid sequences.
File: oo_95375.xlsx Matthew L. Bowser

Supplementary material 5 Read counts by species and samples Data type: occurrences
Brief description: Arthropod occurrence data from the metagenomic analysis expressed as read counts. BIN: BOLD Barcode Index Number. Columns labeled KNWR:Ento:10838–KNWR:Ento:10847 are GUIDs of corresponding specimen records on Arctos.
File: oo_95376.xlsx Matthew L. Bowser

## Figures and Tables

**Table 1.** Sample collection data. Complete collection data including photographs of the sampling sites are available from Arctos. Dates are given in ISO 8601 format.

**Arctos GUID**	**BioSample**	**latitude**	**longitude**	**date**
KNWR:Ento:10838	SAMN04999859	59.96477475	-151.1925941	2015-07-19
KNWR:Ento:10839	SAMN04999860	60.03624489	-151.1865056	2015-07-19
KNWR:Ento:10840	SAMN04999861	59.96580488	-151.2419688	2015-07-19
KNWR:Ento:10841	SAMN04999862	60.08219062	-151.1374512	2015-07-18
KNWR:Ento:10842	SAMN04999863	60.05857897	-151.1845976	2015-07-18
KNWR:Ento:10843	SAMN04999864	60.05961196	-151.2341125	2015-07-18
KNWR:Ento:10844	SAMN04999865	60.10452370	-151.1355072	2015-07-18
KNWR:Ento:10845	SAMN04999866	60.10547999	-151.1805821	2015-07-18
KNWR:Ento:10846	SAMN04999867	59.92113378	-151.2456943	2015-07-19
KNWR:Ento:10847	SAMN04999868	59.94346937	-151.2438328	2015-07-19

**Table 2.** Library composition by country and province/state. Country and province values of NA indicate sequences lacking corresponding geographic data.

**Country**	**Province**	**Number of records**
Canada	Alberta	2
Canada	British Columbia	193,410
Canada	Manitoba	4
Canada	Newfoundland and Labrador	2
Canada	Northwest Territories	1
Canada	Ontario	2
Canada	Prince Edward Island	2
Canada	Quebec	3
Canada	Yukon Territory	35,406
Russia	Chukot Autonomous Okrug	406
Russia	Kamchatka Krai	665
United States	Alaska	6,923
NA	NA	11
**Total**		**236,837**

**Table 3.** Composition of sweep net samples as determined by sight identifications.

**Class**	**Order**	**Family**	**Genus**	**Species**	**Quantity**
Arachnida	Acari				1
	**Total Acari**				**1**
Arachnida	Araneae				2
Arachnida	Araneae	Tetragnathidae	* Tetragnatha *		2
Arachnida	Araneae	Thomisidae	* Misumena *	*Misumena vatia* (Clerck, 1757)	1
	**Total Araneae**				**5**
Insecta	Coleoptera				6
Insecta	Coleoptera	Elateridae			1
Insecta	Coleoptera	Staphylinidae			2
	**Total Coleoptera**				**9**
Insecta	Diptera				135
Insecta	Diptera	Agromyzidae			2
Insecta	Diptera	Bibionidae			1
Insecta	Diptera	Chironomidae			23
Insecta	Diptera	Culicidae			3
Insecta	Diptera	Empididae			45
Insecta	Diptera	Ephydridae			1
Insecta	Diptera	Lauxaniidae	* Lauxania *	*Lauxania shewelli* Perusse & Wheeler, 2000	14
Insecta	Diptera	Phoridae			30
Insecta	Diptera	Pipunculidae			2
Insecta	Diptera	Rhagionidae	* Symphoromyia *		5
Insecta	Diptera	Scathophagidae			4
Insecta	Diptera	Simuliidae			13
Insecta	Diptera	Sphaeroceridae			2
Insecta	Diptera	Syrphidae			1
	**Total Diptera**				**281**
Insecta	Hemiptera				238
Insecta	Hemiptera	Aphididae			39
Insecta	Hemiptera	Cicadellidae			257
Insecta	Hemiptera	Miridae			29
Insecta	Hemiptera	Miridae	* Irbisia *		28
Insecta	Hemiptera	Nabidae	* Nabis *		9
Insecta	Hemiptera	Psyllidae			80
	**Total Hemiptera**				**680**
Insecta	Hymenoptera				27
Insecta	Hymenoptera	Ichneumonidae			9
Insecta	Hymenoptera	Sphecidae			2
Insecta	Hymenoptera	Tenthredinidae			6
Insecta	Hymenoptera	Torymidae	* Torymus *		2
	**Total Hymenoptera**				**46**
Insecta	Lepidoptera				3
	**Total Lepidoptera**				**3**
Insecta	Psocoptera				4
	**Total Psocoptera**				**4**

**Table 4.** Summary of occurrence data from the metagenomic analysis. BIN: BOLD Barcode Index Numbers from matched sequences. *f*: frequency of occurrence, the proportion of all samples in which each taxonomic unit was detected.

**Order**	**Family**	**Species**	**BIN**	***f***
Araneae	Thomisidae	*Misumena vatia*	BOLD:AAA6275	0.1
Coleoptera	Chrysomelidae	*Altica tombacina*	BOLD:AAG3656	0.2
Coleoptera	Elateridae	*Hypnoidus bicolor*	BOLD:AAH2367	0.1
Diptera	Anthomyiidae	Anthomyiidae sp. BOLD:AAG2469	BOLD:AAG2469	0.3
Diptera	Anthomyiidae	*Botanophila relativa*	BOLD:ACG5832	0.1
Diptera	Anthomyiidae	*Botanophila rubrigena*	BOLD:ABX5204	0.1
Diptera	Anthomyiidae	*Delia echinata*	BOLD:ACT6183	0.1
Diptera	Anthomyiidae	*Hylemya variata*	BOLD:AAG2478	0.4
Diptera	Anthomyiidae	*Paradelia brunneonigra*	BOLD:ACB1112	0.1
Diptera	Anthomyiidae	*Pegomya* sp. BOLD:AAG2506	BOLD:AAG2506	0.1
Diptera	Anthomyzidae	*Anthomyza* sp. BOLD:AAL8100	BOLD:AAL8100	0.2
Diptera	Bibionidae	Bibionidae sp. BOLD:ACG6252	BOLD:ACG6252	0.2
Diptera	Chironomidae	*Metriocnemus* sp. BOLD:ACB8808	BOLD:ACB8808	0.1
Diptera	Chironomidae	*Smittia* sp. 16ES	BOLD:AAB0375	0.1
Diptera	Chironomidae	*Smittia* sp. ES12	BOLD:AAB0377	0.1
Diptera	Culicidae	*Aedes pullatus*	BOLD:AAM4536	0.1
Diptera	Empididae	*Empididae* sp. BOLD:AAF9792	BOLD:AAF9792	0.3
Diptera	Fanniidae	*Fannia aethiops*	BOLD:AAM6399	0.5
Diptera	Fanniidae	*Fannia serena*	BOLD:AAG6901	0.1
Diptera	Heleomyzidae	*Suillia convergens*	BOLD:AAV8347	0.1
Diptera	Hybotidae	*Euthyneura* sp. BOLD:AAF9859	BOLD:AAF9859	0.1
Diptera	Lauxaniidae	*Lauxania shewelli*	BOLD:AAH3531	0.4
Diptera	Muscidae	*Coenosia impunctata*	BOLD:AAQ0758	0.5
Diptera	Muscidae	*Hydrotaea militaris*	BOLD:AAG1771	0.3
Diptera	Muscidae	Muscidae sp. BOLD:ACL9946	BOLD:ACL9946	0.1
Diptera	Muscidae	*Myospila meditabunda*	BOLD:AAD7145	0.1
Diptera	Phoridae	*Megaselia diversa*	BOLD:ACX1594	0.2
Diptera	Phoridae	Phoridae sp. BOLD:AAG3234	BOLD:AAG3234	0.1
Diptera	Phoridae	Phoridae sp. BOLD:AAL9069	BOLD:AAL9069	0.1
Diptera	Pipunculidae	*Pipunculus campestris*	BOLD:AAD0917	0.2
Diptera	Pipunculidae	*Pipunculus hertzogi*	BOLD:AAE4793	0.5
Diptera	Pipunculidae	*Tomosvaryella* sp. BOLD:AAG3766	BOLD:AAG3766	0.1
Diptera	Psychodidae	*Psychoda phalaenoides*	BOLD:AAF9317	0.1
Diptera	Rhagionidae	*Symphoromyia* sp. BOLD:AAP6399	BOLD:AAP6399	0.4
Diptera	Scathophagidae	*Scathophaga furcata*	BOLD:ACX4405	0.2
Diptera	Scathophagidae	*Scathophaga suilla*	BOLD:AAN6699	0.1
Diptera	Sciaridae	*Cratyna* sp. BOLD:AAP6470	BOLD:AAP6470	0.1
Diptera	Sciaridae	Sciaridae sp. BOLD:AAH3999	BOLD:AAH3999	0.1
Diptera	Sepsidae	*Sepsis neocynipsea*	BOLD:ABY4960	0.5
Diptera	Simuliidae	*Simulium arcticum* complex	BOLD:AAA8954	0.1
Diptera	Simuliidae	*Simulium venustum* complex	BOLD:AAA4264	0.2
Diptera	Syrphidae	*Hiatomyia* sp. BOLD:AAZ5940	BOLD:AAZ5940	0.1
Hemiptera	Aphididae	*Macrosiphum euphorbiae*	BOLD:AAA6213	0.2
Hemiptera	Cicadellidae	*Balclutha* sp. BOLD:AAG8963	BOLD:AAG8963	0.1
Hemiptera	Cicadellidae	*Boreotettix* sp.		0.1
Hemiptera	Cicadellidae	*Diplocolenus evansi*		0.1
Hemiptera	Cicadellidae	*Empoasca luda*	BOLD:AAG8683	0.1
Hemiptera	Cicadellidae	*Euscelis monodens* sp. nov	BOLD:ACG7815	0.5
Hemiptera	Cicadellidae	*Idiocerus* sp. BOLD:ACB0208	BOLD:ACB0208	0.2
Hemiptera	Cicadellidae	*Latalus tatraensis*		0.1
Hemiptera	Cicadellidae	*Limotettix dasidus*	BOLD:AAG8684	0.1
Hemiptera	Cicadellidae	*Sonronius dahlbomi*	BOLD:AAN8426	0.8
Hemiptera	Cicadellidae	*Twiningia fasciata*		0.1
Hemiptera	Miridae	*Irbisia sericans*	BOLD:AAZ2844	0.1
Hemiptera	Miridae	*Mecomma gilvipes*	BOLD:AAZ6451	0.3
Hemiptera	Miridae	*Salignus tahoensis*	BOLD:AAF9947	0.2
Hemiptera	Psyllidae	*Craspedolepta alaskensis*	BOLD:ACM1279	0.9
Hemiptera	Psyllidae	*Craspedolepta subpunctata*	BOLD:AAV0232	0.3
Hymenoptera	Braconidae	*Microgaster* jft23	BOLD:AAB8447	0.1
Hymenoptera	Ichneumonidae	*Mesochorus prolatus*	BOLD:ACE4725	0.1
Hymenoptera	Ichneumonidae	*Orthocentrinae* sp. BOLD:AAH1521	BOLD:AAH1521	0.1
Hymenoptera	Ichneumonidae	*Polysphincta limata*	BOLD:AAH1739	0.1
Hymenoptera	Tenthredinidae	*Amauronematus fallax*	BOLD:ABU5508	0.1
Lepidoptera	Noctuidae	*Alypia langtoni*	BOLD:AAD5114	0.1
Lepidoptera	Plutellidae	*Plutella hyperboreella*	BOLD:AAC3387	0.1
Lepidoptera	Tortricidae	*Argyrotaenia occultana*	BOLD:AAA2955	0.1
Psocoptera	Caeciliusidae	*Valenzuela flavidus*	BOLD:AAN8447	0.1

**Table 5.** Summary of plant species occurrences. GBIF ID: GBIF (http://www.gbif.org/) taxon identifier. *f*: frequency of occurrence, the proportion of all samples in which each taxonomic unit was detected.

**order**	**family**	**scientific name**	**GBIF ID**	***f***
Apiales	Apiaceae	*Conioselinum chinense* (L.) Britton, Sterns & Poggenb.	3034690	0.1
Apiales	Apiaceae	*Heracleum maximum* Bartr.	3034826	0.3
Asterales	Asteraceae	*Achillea borealis* Bong.	3120086	0.3
Asterales	Asteraceae	*Senecio triangularis* Hook.	3108906	0.1
Caryophyllales	Caryophyllaceae	*Moehringia lateriflora* (L.) Fenzl	3085371	0.1
Dipsacales	Adoxaceae	*Sambucus racemosa* L.	2888723	0.2
Equisetales	Equisetaceae	*Equisetum arvense* L.	7924597	0.4
Equisetales	Equisetaceae	*Equisetum* L.	2687913	0.5
Equisetales	Equisetaceae	*Equisetum sylvaticum* L.	2687929	0.2
Ericales	Ericaceae	*Pyrola asarifolia* Michx.	2888271	0.2
Ericales	Ericaceae	*Vaccinium caespitosum* Michaux	2882860	0.1
Ericales	Ericaceae	*Vaccinium vitis-idaea* L.	2882835	0.1
Ericales	Polemoniaceae	*Polemonium acutiflorum* Willd. ex Roem. & Schult.	2927866	0.1
Ericales	Primulaceae	*Trientalis europaea* L.	3169295	0.1
Fabales	Fabaceae	*Lupinus nootkatensis* Sims	2964525	0.6
Fagales	Betulaceae	*Alnus* Mill.	2876099	0.1
Gentianales	Gentianaceae	*Swertia perennis* L.	5414540	0.2
Gentianales	Rubiaceae	*Galium* L.	2913027	0.2
Geraniales	Geraniaceae	*Geranium erianthum* DC.	2890394	0.6
Lamiales	Orobanchaceae	*Castilleja unalaschcensis* (Cham. & Schltdl.) Malte	3170721	0.4
Liliales	Liliaceae	*Streptopus amplexifolius* (L.) DC.	2752734	0.9
Liliales	Melanthiaceae	*Veratrum viride* Aiton	7575112	0.7
Malpighiales	Salicaceae	*Populus tremuloides* Michx.	3040215	0.1
Malpighiales	Salicaceae	*Salix barclayi* Anderss.	5372597	0.1
Malpighiales	Salicaceae	*Salix* L.	3039576	0.2
Malpighiales	Violaceae	*Viola* L.	2874237	0.1
Myrtales	Onagraceae	*Chamerion angustifolium* (L.) J. Holub	3188783	1.0
Pinales	Pinaceae	*Picea lutzii* Little	5284875	0.1
Poales	Cyperaceae	*Carex macrochaeta* C.A.Mey.	2723223	0.1
Poales	Cyperaceae	*Carex mertensii* J.D.Prescott ex Bong.	2722481	0.2
Poales	Juncaceae	*Luzula parviflora* (Ehrh.) Desv.	2700961	0.2
Poales	Poaceae	*Alopecurus magellanicus* Lam.	4107552	0.1
Poales	Poaceae	*Calamagrostis canadensis* (Michx.) P.Beauv.	2704895	1.0
Poales	Poaceae	*Festuca altaica* Trin.	7720963	0.1
Poales	Poaceae	*Phleum alpinum* L.	2706012	0.1
Poales	Poaceae	*Poa arctica* R.Br.	2704207	0.1
Polypodiales	Athyriaceae	*Athyrium filix-femina* (L.) Roth	5275044	0.5
Polypodiales	Cystopteridaceae	*Gymnocarpium dryopteris* (L.) Newm.	2650832	0.1
Polypodiales	Dryopteridaceae	*Dryopteris expansa* (C. Presl) Fraser-Jenk. & Jermy	5275102	0.6
Ranunculales	Ranunculaceae	*Aconitum delphiniifolium* Hort.Prag. ex Steud.	7994520	0.4
Rosales	Rosaceae	*Rubus arcticus* L.	2992051	0.1
Rosales	Rosaceae	*Rubus idaeus* L.	2993094	0.1
Rosales	Rosaceae	*Rubus* L.	2988638	0.1
Rosales	Rosaceae	*Rubus pedatus* Sm.	2993074	0.2
Rosales	Rosaceae	*Sanguisorba canadensis* L.	3029411	0.7
Rosales	Rosaceae	*Spiraea stevenii* (Schneid.) Rydb.	3026628	0.2
